# Comprehensive Characterization of the Immune Microenvironment of Colorectal and Gastric Signet Ring Cell Cancer

**DOI:** 10.3390/cells15010030

**Published:** 2025-12-23

**Authors:** Jianqing Zhang, Robin Collingwood, Sameer Al Diffalha, Deborah Della Manna, Ravi Kumar Paluri, Haider A. Mejbel, Olumide Gbolahan

**Affiliations:** 1 Department of Radiation Oncology, Heersink School of Medicine, University of Alabama at Birmingham, Birmingham, AL 35294, USA; jianqingzhang@uabmc.edu (J.Z.);; 2 Department of Clinical Pathology and Laboratory Medicine, Perelman School of Medicine, University of Pennsylvania, Pennsylvania, PA 19104, USA; 3 Department of Pathology, Miller School of Medicine, University of Miami, Miami, FL 33136, USA; 4 Department of Hematology and Medical Oncology, Atrium Health Wake Forest Baptist Medical Center, Winston-Salem, NC 27157, USA; 5 Department of Pathology, Emory University School of Medicine and Winship Cancer Institute, Atlanta, GA 30322, USA; haider.a.mejbel@emory.edu; 6 Department of Hematology and Medical Oncology, Emory University School of Medicine and Winship Cancer Institute, Atlanta, GA 30322, USA

**Keywords:** signet ring cell cancer, colorectal cancer, gastric cancer, immune microenvironment, gene expression profiling, IL13RA2

## Abstract

**Highlights:**

**What are the main findings?**

- The immune components of gastric and colorectal SRCCs are distinct from their corresponding non-SRCC counterparts.
- IL13RA2 was identified as a potential biomarker and therapeutic target candidate for colorectal SRCC.

**What are the implications of the main findings?**

- Gastric and colorectal SRCC tumors likely employ immune evasion mechanisms.
- Overall, our descriptive study highlights pathways (IL-13, complement) that may be targeted for drug development in SRCC.

**Abstract:**

The reasons for the aggressive clinical phenotype of signet ring cell carcinoma (SRCC) have not been fully elucidated. Previous studies suggest similarities in the genotype of colorectal and gastric SRCC and a clear distinction from non-SRCC. The immune microenvironments of gastric and colorectal SRCC have not been comprehensively examined. We isolated RNA from formalin-fixed, paraffin-embedded (FFPE) sections of 34 tumor specimens, 10 colorectal SRCC, 24 gastric SRCC, 4 non-SRCC colorectal (CCC), and 3 gastric adenocarcinoma (GCC) samples. The PanCancer Immune Profiling Panel was used to evaluate the expression of 770 immune-related genes. We compared the expression profiles of colorectal and gastric SRCC and non-SRCC adenocarcinoma. We found that the immune-related gene expression profiles (GEPs) of colorectal SRCC (CR-SRCC) and gastric SRCC (G-SRCC) were distinct from the non-SRCC. A total of 127 genes were upregulated and 32 downregulated in CR-SRCC compared to CCC. Only two genes (CCL27 and LAIR2 reached statistical significance (*p*-adj < 0.05)) among the differentially expressed genes in G-SRCC compared to GCC. None of the clinically relevant immune checkpoints were significantly differentially expressed in SRCC vs. non-SRCC. Overall, we noted a relative abundance of CD8+ cells in CR-SRCC and G-SRCC and relative overexpression of genes involved in innate immune response including the complement pathway. Finally, we identified IL13RA2 as a potential biomarker and therapeutic target candidate for CR-SRCC. The immune microenvironments of CR-SRCC and G-SRCC are distinct from non-SRCC. Broadly, both CR-SRCC and G-SRCC are characterized by a complex immune microenvironment that features cytotoxic cells and innate immune activity that may facilitate immune evasion.

## 1. Introduction

Signet ring cell carcinoma (SRCC) is a distinct histologic subtype of adenocarcinoma characterized by an abundance of intracellular mucin that displaces the nucleus towards the periphery to give a signet ring appearance [1]. Adenocarcinoma with this morphology has been described in multiple organ systems but is most prevalent in the gastrointestinal system [1]. About 55% of SRCC are reported in the stomach (where it represents 15–25% of gastric adenocarcinoma) and up to 15% arise from the colon, representing 1% of the incident cases of colon cancer [1]. Despite its relative rarity, SRCC demands attention because of its unique clinical behavior. In comparison to non-signet ring adenocarcinoma of the same organ, it is associated with a more aggressive phenotype. For example, only 12% of colorectal signet ring cell carcinoma (CR-SRCC) present with localized disease (without lymph node involvement), compared to 30% of non-signet ring colorectal adenocarcinoma (CRC). Consequently, 40% of CR-SRCC present with metastatic disease, compared to about 25% of CRC [2,3,4]. There is also a preponderance of poorly differentiated histology in CR-SRCC [5]. Given these characteristics, it is not surprising that signet ring morphology is associated with poor prognosis, independent of disease stage, and other pathologic features [6,7,8].

In gastric cancer, the differences in clinical and pathological characteristics of non-signet ring gastric adenocarcinoma (GC) and gastric SRCC (G-SRCC) are not as stark as in CRC, but the trend of presentation at more advanced stages and with more poorly differentiated disease persists. Further, the prognostic implications of SRCC are not quite as well-defined in gastric cancer [9,10].

Early work suggested that the aggressive phenotype, especially the predilection for peritoneal spread of G-SRCC and CR-SRCC, may be related to a loss of function of proteins involved in cell adhesion. For example, in a study of 1337 SRCC samples involving various organs, attenuation or loss of E-Cadherin was reported in up to 76% of cases. The investigators also reported aberrant β-Catenin expression (a key partner with cadherins for mediating cell adhesion) in 60% of samples [10]. This shared molecular fingerprint and clinical phenotype raised the possibility that G-SRCC and CR-SRCC may have molecular commonalities compared to their non-SRCC counterparts.

This hypothesis was explored by Puccini and colleagues in their review of next-generation sequencing (NGS) data from 8500 samples of CRC and 1100 samples of GC. Approximately one and nine percent, respectively, were SRCC. They concluded that ‘SRCCs harbor similar molecular profile, regardless of the tumor location’. On the other hand, significant differences were observed between SRCCs and non-SRCC [11].

While a significant amount of work has been performed to characterize the molecular profiles of G-SRCC and CR-SRCC, there remains a paucity of information about the immune microenvironment of SRCC, especially for CR-SRCC. It is important to fill this gap in the field, as immune modulating therapies have emerged in the management of multiple malignancies including GC. Most available studies have been limited to assessing the presence of T cells, mainly based on immunohistochemical techniques [12]. These studies provide limited data about the other immune cell types and the potential functional implication of these cells in the immune microenvironment. We therefore designed a study to comprehensively characterize the immune profile of G-SRCC and CR-SRCC. We posited that there would be significant overlap between the immune profile of colorectal and gastric SRCC and that these profiles would be distinct from the corresponding non-SRCC adenocarcinoma.

## 2. Materials and Methods

### 2.1. Patients and Samples

Ten CR-SRCC and twenty-four G-SRCC tumor samples, from adult patients who received treatment at the University of Alabama, Birmingham School of Medicine, were identified. Formalin-fixed paraffin-embedded (FFPE) blocks were retrieved from the archives of the Department of Pathology. We also obtained four CRC and three GC samples each to serve as controls (CCC and GCC, respectively). Microdissection was performed to enrich tumor-rich regions of each specimen. The diagnosis of SRCC, based on the WHO definition of 50% signet ring component, was made by two pathologists (RC and SA). Patient characteristics, including sex and age, are summarized in Table 1. The study was performed under a protocol approved by the Institutional Review Board of the University of Alabama at Birmingham.

### 2.2. Nucleic Acid Extraction and Purification

Total ribonucleic acid (RNA) was extracted and purified from microdissected samples from unstained, 10 μm sections of formalin-fixed paraffin-embedded (FFPE) slides (2–5), following manufacturer’s instruction, with the High Pure FFPET RNA isolation Kit (Roche Diagnostics, Indianapolis, IN, USA). The RNA was further purified and concentrated with the RNA Clean & Concentrator TM-5 (Zymo Research, Irvine, CA, USA). An amount of 350 ng of total RNA was applied to the hybridization of the human Immune Profiling code set (NanoString Technologies/Bruker Spatial Biology, Seattle, WA, USA).

### 2.3. NanoString Assay for Gene Expression Analysis

Gene expression was quantified digitally using an nCounter analysis system (Bruker Spatial Biology, Seattle, WA, USA) with the PanCancer Immune Profiling Panel, a unique 770-plex gene expression panel to measure the human immune response in both liquid and solid cancer types [13]. This panel measures many features of the immune response to facilitate rapid development of clinically actionable gene expression profiles in the context of cancer immunotherapy, including major classes of cytokines and their receptors, enzymes with specific gene families such as major chemokine ligands and receptors, interferons and their receptors, the TNF-receptor superfamily, and the KIR family genes. The nCounter Analysis System is based on a novel digital color-coded barcode technology which allows for direct multiplexed measurement of gene expression from low amounts of mRNA (as low as 25 ng) without need for amplification in a single reaction with high precision and sensitivity [14], and the data were managed and analyzed with nSlover 4.0 basic software and extended with nCounter Advanced Analysis 2.0 following the manufacture’s user manual: https://nanostring.com/wp-content/uploads/MAN-C0019-08_nSolver_4.0_Analysis_Software_User_Manual.pdf (accessed on 9 December 2025). The nCounter Raw data were also channeled to the Rosalind platform for further comparisons: Rosalind analysis. The differential expressed gene sets (DEGs) derived from Nanostring/Rosalind analysis were further imported into the site: CPDB platform for molecular concept-based interaction network analysis. Non-parametric tests were performed because the distribution of expression values did not meet assumptions required for parametric analysis.

### 2.4. Cell Type Profiling Methods for Nanostring nCounter Gene Expression Data

The nCounter human Immune Profiling panel feature genes provide unique cell profiling data to measure the relative abundance of different immune cell types. This 770-plex assay panel contains the following: 109 genes for cell surface markers capable of quantitating 24 different tumor infiltrating immune cell types and populations, 30 genes for commonly studied CT Antigens, over 500 genes for measuring immune response with a special emphasis on checkpoint regulation/signaling, and 40 PanCancer Reference Genes [15]. The abundance of various cell types is estimated using the Cell Type Profiling algorithm developed by the NanoString Bioinformatics team. A cell type’s abundance can be measured as the average log-scale expression of its characteristic genes. The algorithm used to identify appropriate marker genes and exclude badly behaving cell type-specific genes from estimates of cell type abundance is described in detail in the manual (Nano AA User Manual.pdf), as is the permutation test used to derive a *p*-value (QC *p*-value) assessing a cell type’s marker genes. The signatures of the marker genes used to quantify the relative abundance of different cell types across samples are listed (Supplemental Table S1). For each cell type, ROSALIND performs a dynamic selection to identify the subset of signature marker genes that are robust and expressed stably and specifically in given cell types. The score for cell type in any given sample is calculated as the arithmetic mean of the log_2_-transformed normalized expression of all selected marker genes for the cell type, and a T-test was performed between cell types of the samples.

## 3. Results

### 3.1. SRCC Immune Gene Expression Profile Is Distinct from Adenocarcinoma

**Figure 1:**
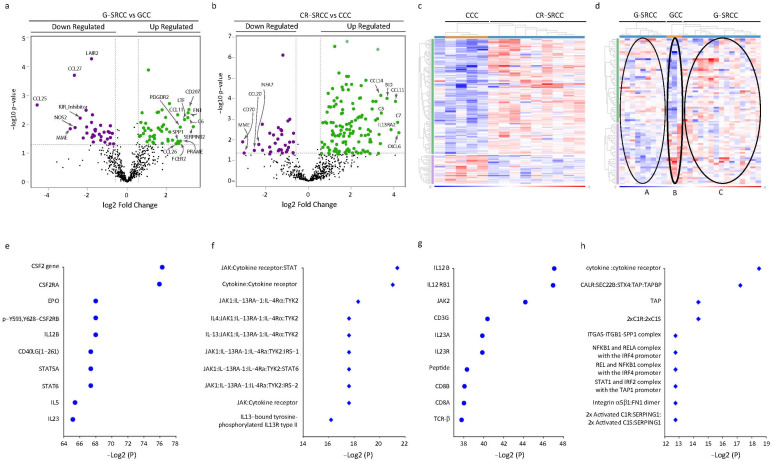
**Immune gene expression in signet ring cell cancer (SRCC) compared to non-signet ring adenocarcinoma.** (**a**) Volcano plot of gene expression in colorectal SRCC (CR-SRCC) compared to colorectal adenocarcinoma (CCC). (**b**) Volcano plot of gene expression in gastric SRCC (G-SRCC) compared to gastric adenocarcinoma (GCC). (**c**) Differential gene expression. The heat map exhibits differential gene expressions between yellow (CCC) and blue (CR-SRCC) samples; blue and red shadings represent lower and higher relative expression levels, respectively. (**d**) Differential gene expressions. The heat map exhibits differential gene expression between yellow (GCC) and blue (G-SRCC) samples; blue and red shadings represent lower and higher relative expression levels, respectively. (**e**,**f**) Top 10 enriched neighborhood–based sets of genes/proteins (**e**) and protein complex-based sets (**f**) in CR-SRCC compared to CCC, representing factors regulating antigen presentation cells like NK and macrophage. (**g**,**h**) Top 10 enriched neighborhood sets of genes/proteins (**g**) and protein complex-based sets (**h**) in G-SRCC compared to GCC, representing factors involved in T cell response.

We hypothesized that a distinct expression profile of immune-related genes would be evident in SRCC as compared to anatomically matched adenocarcinoma. The quality and integrity of the samples were acceptable, as housekeeping genes used for data normalization showed steady expression levels in the samples (Supplemental Figure S1a–f). One hundred and twenty-seven (127) genes were significantly upregulated, and thirty-two genes were downregulated in CR-SRCC compared to CCC (adjusted *p* < 0.05) (Figure 1a, Supplemental Figure S2a). Table 2 summarizes the top differentially expressed genes (DEG) (more than 1.5 of log2-fold change) in CR-SRCC compared to CCC. We explored the relative expression of clinically relevant immune checkpoints including CTLA4, PD-1, PDL-1, and LAG, but none of these were represented among the top 20 DEG in CR-SRCC compared to CCC. However, there was at least a 10-fold linear increase in the expression of CXCL6, C7, CCL11, IL13RA2, BID, C3, and SELE in CR-SRCC samples compared to CCC. Conversely, CD70, MME, CCL20, and IFNA7 expression were 5-fold lower in CR-SRCC (Table 2). In contrast to the data in CR-SRCC and CCC, when using the same adjusted significance threshold (*p* < 0.05), there were fewer DEG in G-SRCC compared to GCC. Forty-seven genes were overexpressed while thirty-four were under-expressed (Figure 1b, Supplemental Figure S2b). There was more than 5-fold higher expression of SERPINB2, CD207, FN1, C6, LTF, PRAME, FCER2, SPP1, and CCL11 in G-SRCC relative to GCC. However, these did not reach statistical significance (Table 3). Similarly, MME, NOS2A, and KIR-IN were expressed 5-fold at lower levels in G-SRCC compared to GCC. CCL27 and LAIR2 were expressed at significantly lower levels in G-SRCC relative to GCC (Table 3). As was the case in colon cancer, no clinically relevant immune checkpoint genes were represented among the top 20 DEG in G-SRCC relative to GCC.

Exploratory analysis (unsupervised clustering) suggested distinct gene expression patterns for CR-SRCC and CCC (Figure 1c). Further, there was a suggestion of two subsets of G-SRCC (Figure 1d). The tumors that segregated into Cluster A shared lower differential-expressed gene sets, and Cluster C shared higher differential-expressed gene sets compared to GCC.

To capture the potential functional consequences of gene expression profiles, these data were aggregated into 21 functionally distinct biomarkers involved in biological processes (Supplemental Figure S3). As expected, there was differential enrichment in each functional pathway among the tumor samples. The expression of genes that correlate with interleukins, cytokines, cytotoxicity, antigen-processing, NK cell, pathogen defense, and chemokine were much lower in CR-SRCC, while expression of genes involved in CT antigen, macrophage function, TNF Superfamily, transporter function, TLR, microglial functions, adhesion, leukocyte function, and complement were more enriched in CR-SRCC (Supplemental Figure S4). Expression of genes involved in B cell, senescence, T cell, cell cycle, T cell function, cell function, and regulation tended to be similar between CR-SRCC and G-SRCC (Supplemental Figure S4).

Neighborhood-based gene set analysis suggested distinct interacting genes and proteins. CSF2, CSF2RA, and EPO were the most significant hits in CR-SRCC compared to CCC (Figure 1e), while IL12B, IL12RB1, and JAK2 were the most significant hits in G-SRCC compared to GCC (Figure 1g). The protein complex-based sets analysis suggested that SRCC differential gene expression profiles compared to non-SRCC controls favored cytokine–cytokine receptor interaction. However, 7 of the top 10 enriched interaction pathways in CR- SRCC related to IL13/IL13R (Figure 1f), and TAP/TAPBP were the top 3 of 7 picks in G-SRCC (Figure 1h).

### 3.2. Colorectal and Gastric SRCC Show Distinct Immune-Related Gene Expression Profiles

**Figure 2:**
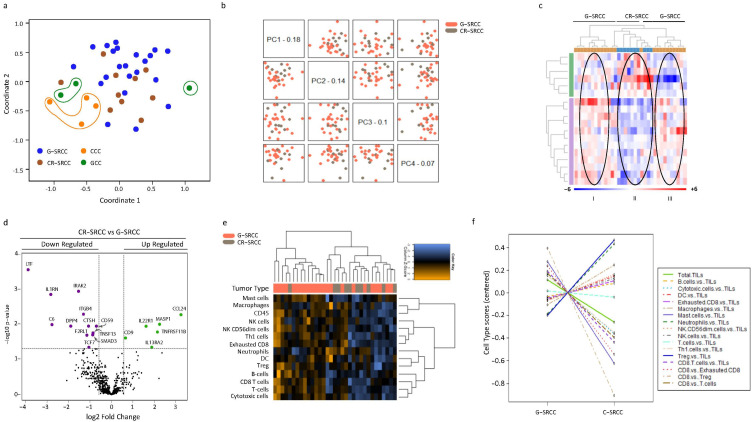
**Summary of immune-related gene expression patterns in CR-SRCC and G-SRCC.** (**a**) Multidimensional scaling plot (MDS) of the pattern of proximity (similarities and distances) among the different cancer subtypes. (**b**) Principal component analysis of gene expression data. Each dot represents a patient sample. Colorectal and gastric signet ring cell cancer represented as gray and pink dots, respectively. (**c**) Heat map clustering of gene expressions in colorectal (blue) and gastric (orange) signet ring cell cancer. Each column represents one patient sample, and a row represents a gene. Overexpressed gene-based normalized counts are red, while blue indicates under expression. (**d**) Volcano plot of gene expression in CR-SRCC compared to G-SRCC. (**e**) Heatmap showing raw abundance of cell types in samples of colorectal SRCC (gray) compared to gastric SRCC (orange). Yellow indicates high abundance; blue indicates low abundance. (**f**) A trend plot of the expression of different gene sets (signatures) in gastric and colorectal SRCC. Underexpressed gene sets extend below the ‘0’ locus and overexpressed sets extend above ‘0’.

We compared the immune-related gene expression profiles of CR-SRCC and G-SRCCC. We hypothesized that there would be significant overlaps in gene and gene set expression patterns between these two SRCC sub-types. A multidimensional scaling plot of the patterns of proximities among the different anatomic and histological subtypes is represented in Figure 2a. The CR-SRCC and G-SRCC gene expression patterns were non-segregated but distinctly separate from colorectal (CCC) and gastric adenocarcinoma (GCC). In addition, the principal component analysis (PCA) of gene expression data was performed with plotting across four components. There was no significant separation between CR-SRCC and G-SRCC. (Figure 2b). A heatmap generated via unsupervised hierarchical analysis to visualize expression patterns of CR-SRCC vs. G-SRCC (Figure 2c) segregated the samples into three main clusters: two G-SRCC subclusters (I and III) and a distinct C-SRCC cluster (Cluster II) positioned between them (II). Regarding gene expression levels, Cluster I showed relatively lower expression of both upregulated and downregulated genes compared to CR-SRCC samples in Cluster II, while Cluster III was characterized by relatively higher expression of genes compared to CR-SRCC (Figure 2c,d; Supplemental Figure S2c).

There was significantly lower relative expression (adjusted *p*-value < 0.005) of LTF, IL1RN, C6, PTGDR2 DPP4, IRAK2, and ITGB4 in CR-SRCC compared to G-SRCC. Conversely, CCL24, MASP1, TNFRSF11B, and IL13RA2 were relatively highly expressed in CR-SRCC compared to G-SRCC (adjusted *p*-value < 0.005, Figure 2d, Supplemental Figure S2c). Table 4 summarizes the top 20 differentially expressed genes in CR-SRCC relative to G- SRCC. Further analysis of clinically relevant immune checkpoints revealed significant relative expression of PD-L1 in G-SRCC compared to CR-SRCC (1.89-fold, *p* < 0.0001). There was no significant difference in relative expression of PD-1, LAG3, CTLA4, TIGIT, and TIM3 between G-SRCC and CR-SRCC (Supplemental Figure S5).

### 3.3. Cytotoxic T Cells Predominate in the Colorectal and Gastric SRCC Microenvironment

We estimated the absolute and relative abundance of different immune cell populations in the tumor samples based on gene expression patterns corresponding to well-characterized phenotypes of immune cells.

**Figure 3:**
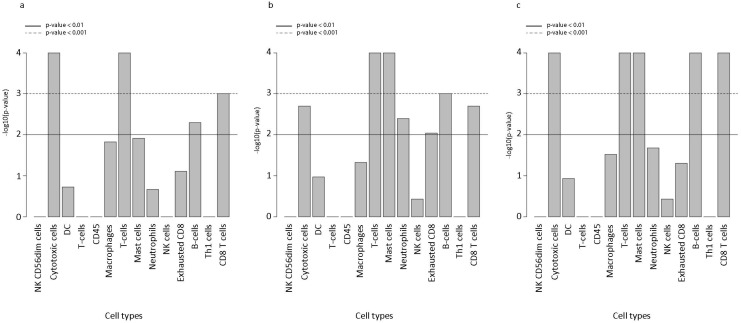
Relative abundance of immune cell populations in tumor samples based on genes characteristic of cell types. (**a**) Relative abundance of immune cells in colorectal signet ring cell cancer (CR-SRCC) compared to colorectal adenocarcinoma (CCC). (**b**) Relative abundance of immune cells in gastric SRCC (G-SRCC) compared to gastric adenocarcinoma (GCC). (**c**) Relative abundance of immune cells in colorectal SRCC (CR-SRCC) compared to gastric SRCC (G-SRCC).

Expression signatures indicated the presence of a cellular milieu composed of cytotoxic cells, T cells (including CD8+ T cells), mast cells, neutrophils, B cells, NK cells, exhausted CD8 T cells, Th1 cells, Treg, CD45, macrophages, and NK CD56dim cells. Overall, there was relatively low expression of CD45 in SRCC (Figure 3). Nevertheless, there was significantly higher relative abundance of signatures for cytotoxic cells (in general) and T cells in CR- SRCC compared to CCC (*p* < 0.001). CD8+ T cell and B cell signatures were relatively more abundant in CR-SRCC compared to CCC (*p* < 0.01), but this was not the case for neutrophil, macrophage, dendritic cell or NK cell signatures (Figure 3a). Similarly, G-SRCC was characterized by higher levels of expression signatures corresponding to cytotoxic cells, including T cells, mast cells, and B cells (*p* < 0.01). In addition, neutrophil and exhausted CD8+ T cell signatures were relatively more abundant in G-SRCC compared to GCC (*p* < 0.05). Finally, gene signatures suggested lower levels of dendritic cells (DC), regulatory T cells (Treg), NK cells, and Th1 cells in both C-SRCC and G-SRCC compared to adenocarcinoma (Figure 3a,b).

**Figure 4:**
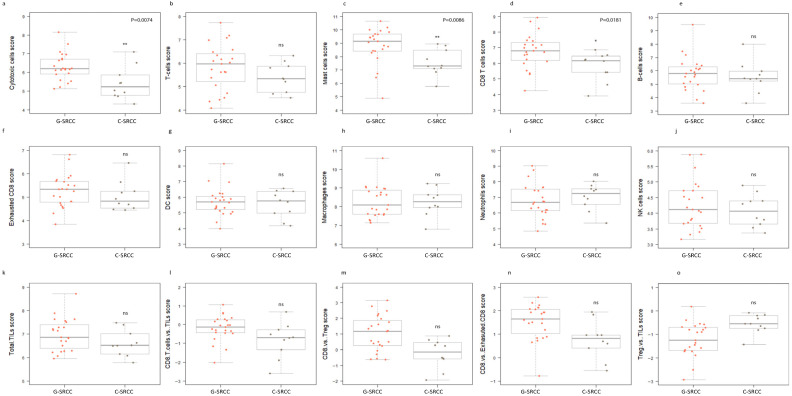
**Measurement of immune cell populations.** (**a**) Relative abundance of cytotoxic cells in G-SRCC compared to CR-SRCC. (**b**) Relative abundance of T cells in G-SRCC compared to CR-SRCC. (**c**) Relative abundance of mast cells in G-SRCC compared to CR-SRCC. (**d**) Relative abundance of CD8+ T cells in G-SRCC compared to CR-SRCC. (**e**) Relative abundance of B cells in G-SRCC compared to CR-SRCC. (**f**) Relative abundance of exhausted CD8 T cells in G-SRCC compared to CR-SRCC. (**g**) Relative abundance of DC in G-SRCC compared to CR-SRCC. (**h**) Relative abundance of macrophage in G-SRCC compared to CR-SRCC. (**i**) Relative abundance of neutrophils in G-SRCC compared to CR-SRCC. (**j**) Relative abundance of NK cells in G-SRCC compared to CR-SRCC. (**k**) Total TIL score in G-SRCC compared to CR-SRCC. (**l**) Relative abundance of CD8 vs. T cells in G-SRCC compared to CR-SRCC. (**m**) Relative abundance of CD8 vs. Treg cells in G-SRCC compared to CR-SRCC. (**n**) Relative abundance of CD8 vs. exhausted CD8 T cells in G-SRCC compared to CR-SRCC. (**o**) Relative abundance of Treg vs. TILs in G-SRCC compared to CR-SRCC. For statistical significance, * *p* < 0.05, ** *p* < 0.01, ns indicates not significant.

We then estimated the relative abundance of immune cell signatures in G-SRCC compared with CR-SRCC. Again, the SRCC microenvironment demonstrated low levels of CD45 + ve signatures. However, the G-SRCC microenvironment was enriched for cytotoxic cell signatures: T cell, including CD8+ T cell, mast cell, and B cell (*p* < 0.0001) (Figure 3c). A heatmap of predicted cell types was in line with this, suggesting a relative abundance of adaptive immunity-related cells in G-SRCC samples compared to CR-SRCC (Figure 2e–f). Overall, the median levels of cytotoxic cell signatures. T cell, mast cell, B cell, CD8+ T cell, and exhausted CD8+ T cell, were higher (Figure 3c and Figure 4a–f), while DC, macrophage, neutrophils, NK cell, and exhausted CD8+ T cell signatures were equal or lower compared to CR-SRCC (Figure 4g–o). The relative abundance of TILs, CD8+ T cells vs. total T cells, CD8+ T cells vs. Treg, and CD8+ T cells vs. exhausted CD8+ T cells were higher in G-SRCC (Figure 4l–n) while the Treg vs. TILs was higher in CR-SRCC (Figure 4o).

## 4. Discussion

We examined the immune microenvironments of the signet ring subtypes of gastric and colorectal adenocarcinoma based on gene expression analysis and immune Cell Type Profiling. We sought to comprehensively characterize the immune profile of SRCC and determine whether there were differences in the immune profile of SRCC relative to non-SRCC adenocarcinoma. We found a striking difference in the gene expression profile of SRCC, particularly colorectal SRCC, compared to non-SRCC adenocarcinoma. Furthermore, despite low overall leukocyte infiltrate, we found a relative abundance of cytotoxic cell signatures in CRC and gastric SRCC compared to non-SRCC adenocarcinoma. This descriptive analysis provides a global view of the immune microenvironment of gastric and colorectal SRCC.

The distinct immune transcript profile of gastric and colorectal SRCC compared to non-SRCC noted in this study extends the narrative that SRCC is a separate entity from non-SRCC adenocarcinoma of the same tissue origin. Previous work based on comprehensive next-generation sequencing data observed significant differences in the genotypes of CR-SRCC and G-SRCC compared to non-SRCC adenocarcinoma [11]. In our study, we describe an immunotype that tracks the clinical phenotypes of colorectal and gastric SRCC. For example, we note that while CR- SRCC showed several significant DEG relative to CCC, the relative difference in immune-related DEG was more modest when we compared gastric SRCC to GCC. As mentioned previously, while there is consensus about the more aggressive nature of CR-SRCC vs. CCC, the data are less clear with G-SRCC vs. GCC [10]. This may be explained by the different microenvironments that spur the development of colorectal and gastric cancer. An immune inflamed subtype has been described within the general population of gastric cancer [16], and in clinical practice, ICI is effective in the management of advanced gastric cancer that presents with a PDL-1 combined proportion (CPS) score of at least 1%. This represents about 75% of advanced gastric cancer (and about 60% with CPS 5% or more) and suggests an immune-rich microenvironment in most gastric cancer [17,18]. This is in sharp contrast with advanced colorectal cancer, where only about 5% of patients may benefit from ICI (MMR deficient or MSI-H) [19], reflecting an ‘immune-cold’ microenvironment in the majority of colorectal cancer. Given the different immunologic backgrounds in the colon and the stomach, it is easy to appreciate how a relative difference in the immune microenvironment of SRCCs compared to non-SRCC would be more readily discernible in colorectal cancer (relatively cold baseline) compared to gastric cancer (relatively warm baseline).

Notwithstanding, our results suggest a T cell-skewed microenvironment with immune evasion mechanisms at play in SRCC compared to non-SRCC adenocarcinoma. This is borne out by the relative abundance of exhausted CD8+ cell signatures, particularly in the gastric SRCC microenvironment (and, to a lesser extent, in CR-SRCC). Further, in colorectal SRCC, relative overexpression of complement factors C7 and C3, chemokines (CXCL6, CCL11, CCL14), and mast cell markers (FCER1A, CMA1) suggest innate immune activity, and overexpression of FCER1A, CMA1, and IL13RA2 may suggest Th2-skewed immunity or allergic inflammation. The relative overexpression of IL13RA2 and underexpression of CXCL11 and IFNA7 also imply tumor-related chronic inflammatory suppressive TME which would support immune evasion. Relatively high complement component expression (C3 and C7) along with enriched neighborhood gene sets for angiogenic cascade factors CSF2 (GM-CSF), CSF2RA, and EPO also add to the narrative of a potentially suppressive microenvironment [20,21,22].

While an immunosuppressive microenvironment has been described by several investigators in G- SRCC, the data are limited in CR-SRCC [23,24]. Alvi and colleagues describe a hypermethylated cohort of CR-SRCC (N = 44). This cohort was characterized by a high infiltration of CD3+ T cells. They also observed a trend towards high PDL expression in this cohort [25]. Of note, almost half (*n* = 20) of CR-SRCC in this cohort was MSI-H, and 75% of these were hypermethylated. As such, their study was enriched for MSI-H cancer which is associated with a PDL-1 + ve, T cell-enriched tumor microenvironment [26,27]. It is, therefore, not surprising that they report an immune-suppressive environment with an increased expression of PDL-1 + cells. Although we did not uncover PDL-1 overexpression in CR-SRCC, our study generally aligns with this characterization of the CR-SRCC immune microenvironment with a relative abundance of effector cytotoxic cells and overexpression of genes that play a role in immune suppression. Similarly, An et al. showed increased CD3+ and CD8+ cells in CR-SRCC compared to non-SRCC adenocarcinoma [28]. They also failed to show increased PDL-1 expression in CR-SRCC relative to adenocarcinoma.

We also uncovered a theme of an immune-suppressive microenvironment in gastric SRCC, complementing the data available in the literature. Despite relatively low levels of CD45+ cell signature, there were relatively higher levels of CD8+ T cell signature in G-SRCC compared to adenocarcinoma. In addition, there was a preponderance of exhausted CD8+ T cell signature. This is in line with single-cell profiling experiments published by Chen and colleagues, who described an immunologically quiescent microenvironment in gastric SRCC, characterized by a relative paucity of CD4+ CD8+ T cells and a preponderance of exhausted CD8+ T cells [29]. An immune-suppressive microenvironment of gastric SRCC was also suggested by data from Zhao et al. In their single-cell RNA sequencing analysis, they reported that gastric SRCC had ‘high immune escape capabilities compared to moderate and poorly differentiated adenocarcinoma cells and infiltration with follicular B cells’ [30].

This study also suggests enhanced activity of innate immune components in gastric SRCC relative to non-SRCC adenocarcinoma. Complement protein C6 was one of the top upregulated genes in our gastric SRCC cohort. This, along with enriched neighborhood genes for IL2, interferon gamma (IFNG), and IL12, augment the argument that innate responses may play a significant role in the immune microenvironment of gastric SRCC compared to gastric adenocarcinoma. Activation of the complement cascade in G-SRCC [23] has been demonstrated by proteomic profiling. Interestingly, gene set analysis (Supplemental Figure S4) suggests that complement function may be similarly important in CR-SRCC, and DEG data showed that C3 and C7 were among the top overexpressed genes in CR-SRCC. Several groups have shown that complement activation aids cancer progression and promotes immune evasion [22,31,32,33]. This may contribute to the poor prognosis of SRCC. To the best of our knowledge, ours is the first data set suggesting a potential role for complement activation in the microenvironment of CR-SRCC.

Another objective of our study was to uncover potential candidates for drug development. IL13RA2 is a cancer testis antigen that has previously been reported in colorectal cancer and gastric cancer (among many other tumor types) [34,35]. Overexpression in CRC is associated with an aggressive clinical phenotype and poor prognosis. In our CR-SRCC cohort, IL13RA2 was expressed at 15-fold higher levels than CCC. Further, neighborhood protein complex-based set interactions suggested a prominent role for IL13/IL13 receptor interactions in CR-SRCC. This suggests that IL13 signaling may play an important role in CR-SRCC pathogenesis. As such, IL13RA2 may serve as a candidate biomarker for CRC-SRCC and may be a potential target for drug development for CR-SRCC [36]. Based on this, the next steps for this study include validating IL13RA2 overexpression in CR-SRCC over CCC by immunohistochemistry, determining its role in CRC-SRCC pathogenesis, and developing an antibody drug conjugate targeting IL13RA2.

This study has several important limitations. The rarity of SRCC (particularly CR-SRCC) substantially limited the feasible sample size for this retrospective single-institution study. The limited number of cases affects statistical robustness and may impact generalizability of the findings. This constraint was likely highlighted in the G-SRCC vs. GCC comparisons where many genes showed increased linear fold expressions but did not reach statistical significance. This pattern is consistent with limited statistical power as a plausible contributor because, with small cohorts, even when fold changes may be biologically meaningful, the ability to satisfy false discovery criteria is markedly reduced.

A second limitation is the heterogeneity of the analyzed tumors with respect to disease stage. For gastric SRCC, existing data suggest that clinical phenotype varies by stage at presentation (early vs. late). It is therefore reasonable to expect that the immune microenvironment may also differ by stage, although this has not been systematically defined in SRCC. Because our cohort included tumors from multiple stages and lacked enough to stratify by stage, the analysis may have potentially averaged out stage-specific immune signals, masking potentially meaningful patterns.

Further, MMR status represents a significant potential confounder in interpreting immune microenvironment data for gastrointestinal malignancies. Due to the retrospective nature of this study, we did not have information about the MMR status of all the G-SRCC samples, and 50% of the CR-SRCC samples lacked MMR characterization. Given that MMR-deficient tumors exhibit a highly T cell-enriched microenvironment, the absence of complete MMR data limits our ability to distinguish MMR-driven immune signatures from those that are intrinsic to SRCC biology. This is especially important for CR-SRCC, where prior studies have reported a disproportionately high rate of MMR deficiency, and without complete annotation in our study, some immune features observed could reflect this underlying biology rather than SRCC histology alone.

In addition, the NanoString platform generates bulk gene expression data and does not provide single-cell resolution. Consequently, all immune cell subsets and functional states described in this study represent inferred signatures rather than direct cellular quantification. This limitation should be considered when interpreting inferred cell states (as we have considered) including, for example, signatures suggestive of CD8+ T cell exhaustion.

Finally, this study is exploratory, and the cohort was underpowered to evaluate correlations between gene expression patterns and clinical outcomes like survival or recurrence. Although the data suggests IL13RA2 as a potential biomarker and drug development candidate, mechanistic conclusions cannot be drawn. Further work will require protein-level validation with larger sample sizes and more rigorous in vitro and in vivo modeling to determine whether IL13RA2 plays a biologically actionable and clinically relevant role in SRCC. These efforts are planned for the next phase of the research work.

## 5. Conclusions

The immune microenvironments of gastric and colorectal SRCC are distinct from non-SRCC gastric and colorectal adenocarcinoma. Broadly, both CRC-SRCC and G-SRCC are characterized by an immune-suppressive environment that is enriched for cytotoxic cells. Complement proteins may play a role in that microenvironment and may contribute to immune evasion and the poor prognosis of SRCC. If confirmed, IL13RA2 may serve as both a biomarker and a therapeutic target for CRC-SRCC.

## Figures and Tables

**Table 1:** Clinicopathologic characteristics (at diagnosis) of patients whose tumor samples were analyzed.

Clinical Characteristics		CR-SRCC (*n* = 10)	G-SRCC (*n* = 24)
**Gender**	Male	5 (50%)	13 (54%)
	Female	5 (50%)	11 (46%)
**Age**	Mean (years)	64.2	66.3
	Range (years)	39–83	46–86
**Tumor Location**	Proximal	5 (50%)	7 (29%)
	Distal	5 (50%)	14 (71%)
**Tumor Differentiation**	Well	-	-
	Moderate	-	1 (4%)
	Poor	9 (90%)	22 (92%)
	Not Reported	1 (10%)	1 (4%)
**Mismatch Repair (MMR) Protein Status**	MMR-Deficient	1 (10%)	-
	MMR-Proficient	4 (40%)	-
	Unknown	5 (50%)	24 (100%)
**Stage—Tumor (T)**	4	5 (50%)	9 (38%)
	3	5 (50%)	7 (29%)
	2	-	5 (21%)
	1	-	3 (13%)
**Stage—Node (N)**	3	7 (70%)	2 (8%)
	2	3 (10%)	9 (38%)
	1	-	9 (38%)
**Stage—Metastasis (M)**	1	2 (20%)	1 (4%)

CR-SRCC, colorectal signet ring cell cancer; G-SRCC, gastric signet ring cell cancer.

**Table 2:** Top 20 differentially expressed genes in colorectal signet ring cell cancer compared to colorectal adenocarcinoma.

Gene Symbol	log_2_-Fold Change	Linear Fold Change	*p*-Value	*p*-adj
Overexpressed				
CXCL6	4.23	18.77	0.000484	0.004431
C7	4.18	18.09	0.000105	0.001398
CCL11	4.07	16.85	0.000005	0.000134
IL13RA2	3.87	14.57	0.000317	0.003164
BID	3.68	12.80	0.000003	0.000095
C3	3.41	10.65	0.000026	0.000496
CCL14	3.39	10.46	0.000002	0.000068
SELE	3.37	10.31	0.002438	0.015140
FCER1A	3.31	9.90	0.000651	0.005375
CMA1	3.28	9.70	0.010342	0.044597
Underexpressed				
CD70	−3.08	−8.43	0.001809	0.012369
MME	−3.01	−8.04	0.009847	0.043288
CCL20	−2.53	−5.78	0.006342	0.032281
IFNA7	−2.32	−5.00	0.002771	0.016601
HLA-DRB3	−2.16	−4.46	0.000143	0.001732
CXCL11	−2.15	−4.43	0.006180	0.031830
IL26	−1.81	−3.51	0.003810	0.022233
TNFRSF12A	−1.81	−3.50	0.000548	0.004723
ELANE	−1.81	−3.50	0.011381	0.047855
CT45A1	−1.76	−3.40	0.008076	0.037967

**Table 3:** Top 20 differentially expressed genes in gastric signet ring cell cancer compared to gastric adenocarcinoma.

Gene Symbol	log_2_-Fold Change	Linear Fold Change	*p*-Value	*p*-adj
Overexpressed				
SERPINB2	3.37	10.33	0.012025	0.258465
CD207	3.15	8.85	0.003043	0.223443
FN1	3.10	8.60	0.003961	0.223443
C6	3.09	8.53	0.005833	0.240869
LTF	2.92	7.59	0.006847	0.257518
PRAME	2.71	6.56	0.038177	0.349486
PTGDR2	2.71	6.53	0.012507	0.258465
FCER2	2.63	6.19	0.045733	0.373031
SPP1	2.59	6.00	0.038717	0.349486
CCL11	2.57	5.95	0.004668	0.225900
Underexpressed				
MME	−2.89	−7.43	0.014220	0.258465
CCL27	−2.69	−6.43	0.000197	0.044501
NOS2A	−2.65	−6.27	0.012858	0.258465
KIR_INHIBITING_SUBGROUP_2	−2.39	−5.23	0.006048	0.240869
CD70	−2.23	−4.70	0.029734	0.304996
IFNA7	−2.09	−4.25	0.003237	0.223443
NCR1	−2.06	−4.16	0.012254	0.258465
IFNG	−2.05	−4.13	0.020150	0.271510
LAIR2	−1.81	−3.51	0.000053	0.035803
GAGE1	−1.81	−3.51	0.008518	0.258465

**Table 4:** Differentially expressed genes in colorectal signet ring cell cancer compared to gastric signet ring cell cancer.

Gene Symbol	log_2_ Fold Change	Linear Fold Change	*p*-Value	*p*-adj
Overexpressed				
CCL24	3.25	9.51	0.000040	0.005406
MASP1	2.25	4.76	0.000090	0.010188
TNFRSF11B	2.15	4.43	0.000301	0.016978
IL13RA2	1.88	3.69	0.001201	0.046690
IL22RA1	1.62	3.07	0.000189	0.011639
CD9	0.66	1.58	0.000594	0.025137
C4BPA	1.41	2.66	0.041827	0.247267
CEACAM6	1.28	2.43	0.032559	0.229828
TNFSF4	1.24	2.36	0.004432	0.081094
NEFL	1.21	2.31	0.006537	0.108424
Underexpressed				
LTF	−3.88	−14.77	0.000000	0.000277
IL1RN	−2.83	−7.10	0.000006	0.001420
C6	−2.77	−6.84	0.000108	0.010419
DPP4	−1.90	−3.72	0.000159	0.011509
IRAK2	−1.54	−2.91	0.000003	0.001154
ITGB4	−1.30	−2.47	0.000031	0.005236
F2RL1	−1.14	−2.21	0.000464	0.020946
CTSH	−1.07	−2.09	0.000170	0.011509
TCF7	−1.05	−2.07	0.001241	0.046690
SMAD3	−0.87	−1.83	0.000424	0.020495
TNFSF13	−0.86	−1.81	0.000347	0.018054
CD59	−0.70	−1.63	0.000155	0.011509

## Data Availability

The data generated for this study is available upon request from the first and corresponding authors.

## Supplementary Materials

The following supporting information can be downloaded at: https://www.mdpi.com/article/10.3390/cells15010030/s1, Figure S1: Expression of housekeeping genes used for data normalization; Figure S2: Differentially expressed genes (DEG) in the comparisons of CR-SRCC, G-SRCC, CCC, and GCC; Figure S3: Heatmap of Nanostring Gene Set Variation Analysis (GSVA). Samples CR-SRCC, G-SRCC, CCC, and GCC; Figure S4: Comparison of Nanostring annotated Gene Set Analysis; Figure S5: Comparison of representative mutated genes involved in cancer (Panel A) and representative ICB target gene set (Panel B). Table S1: Marker genes for quantifying the relative abundance of different immune cell types.
