# Supplementary material for: Comprehensive Characterization of the Immune Microenvironment of Colorectal and Gastric Signet Ring Cell Cancer
Source: Cells. 2025 Dec 23;15(1):30. doi: 10.3390/cells15010030 (PMC12785751; doi:10.3390/cells15010030)
Supplement: Supplementary file 1 [file cells-15-00030-s001.zip › GI SRCC Supplemental Table S1.pdf]

Supplemental Table S1: Marker genes for quantifying the relative abundance of different immune cell types

Cell Type	Gene Symbol
B Cells	BLK. CD19, FAM30A, FCRL2, MS4A1, PNOC, SPIB, TCL1A, TNFRSF17
CD45	PTPRC
CD8 T Cells	CD8A, CD8B
Cytotoxic Cells	CTSW, GNLY, GZMA, GZMB, GZMH, KLRB1, KLRD1, KLRK1, NKG7, PRF1
Dendritic Cells	CCL13, CD209, HSD11B1
Exhausted CD8	CD244, EOMES, LAG3, PTGER4
Macrophages	CD163, CD68, CD84, MS4A4A
Mast Cells	CPA3, HDC, MS4A2, TPSAB1/B2
NK CD56dim Cells	IL21R, KIR2DL3/4, KIR3DL1/2
NK Cells	NCR1, XCL1/2
Neutrophils	CEACAM3, CSF3R, FCAR, FCGR3A/B, FPR1, S100A12, SIGLEC5
T Cells	CD3G, SH2D1A, CD6, CD3E
Th1 Cells	TBX21
Tregs	FoxP3
