# Supplementary figures and images for: Comprehensive Characterization of the Immune Microenvironment of Colorectal and Gastric Signet Ring Cell Cancer
Source: Cells. 2025 Dec 23;15(1):30. doi: 10.3390/cells15010030 (PMC12785751; doi:10.3390/cells15010030)

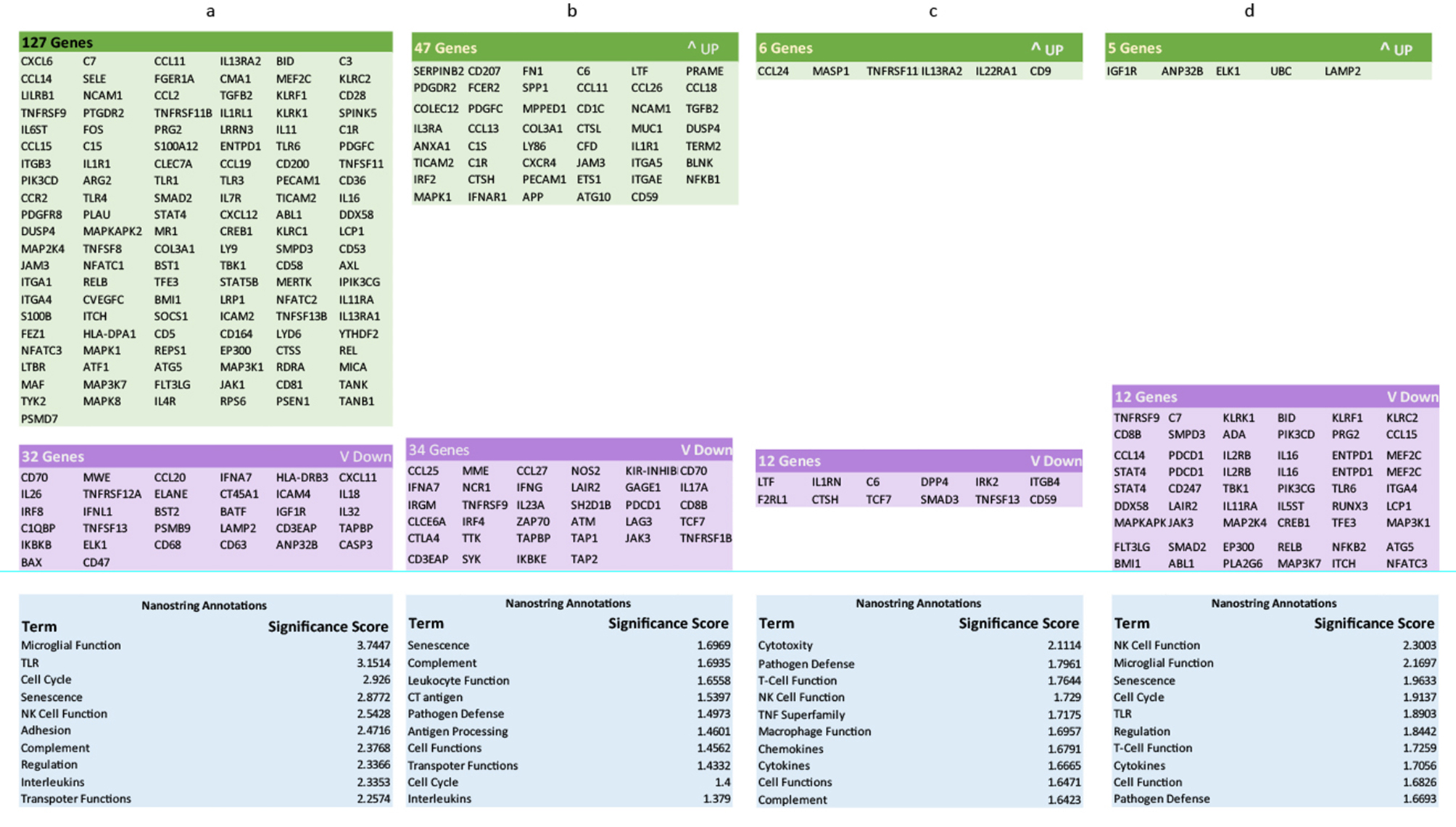

Supplement: Supplementary file 1 [file cells-15-00030-s001.zip › Supplemental Figure -S2_10022025 (1).jpg]

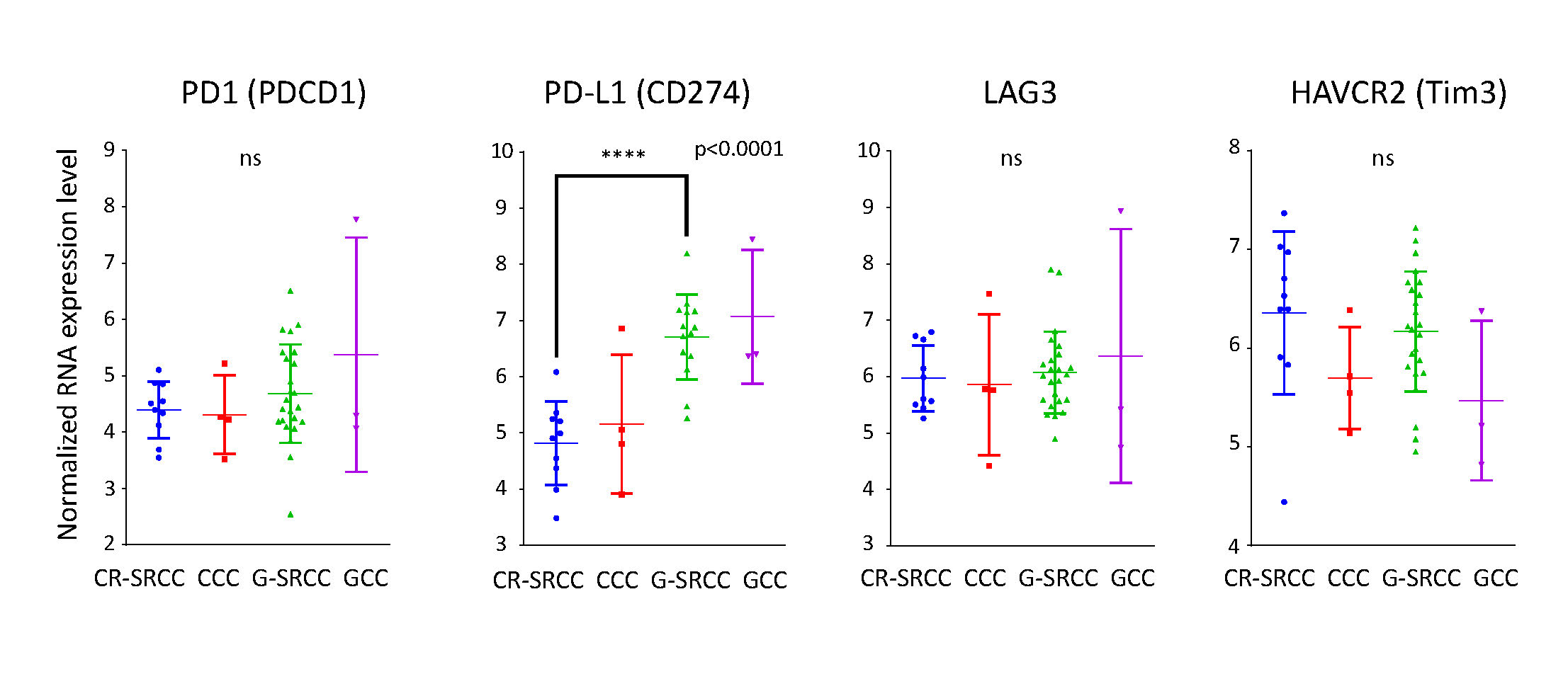

Supplement: Supplementary file 1 [file cells-15-00030-s001.zip › Supplemental Figure S5.jpg]

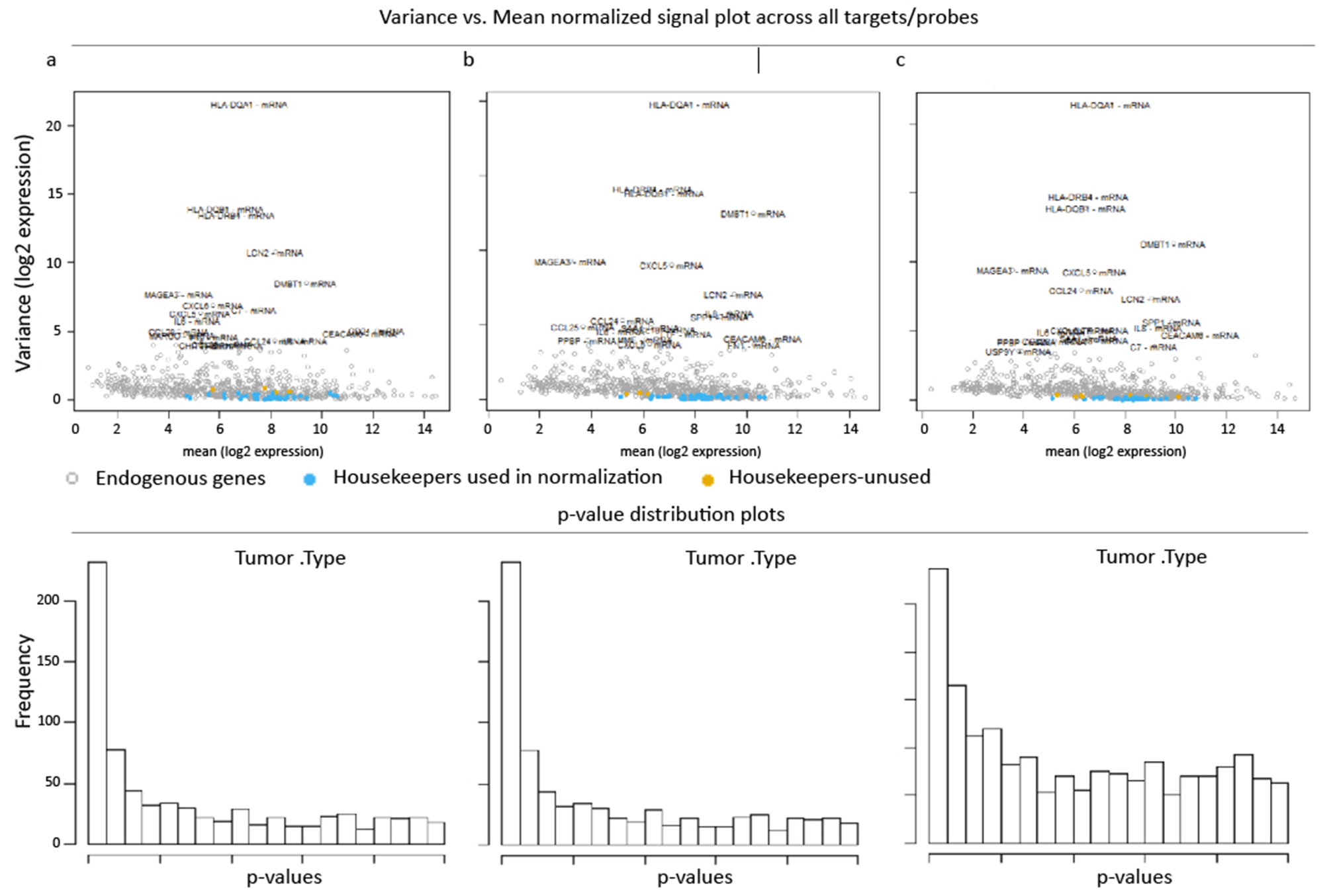

Supplement: Supplementary file 1 [file cells-15-00030-s001.zip › Supplemental Figure-S1_10022025 (1).jpg]

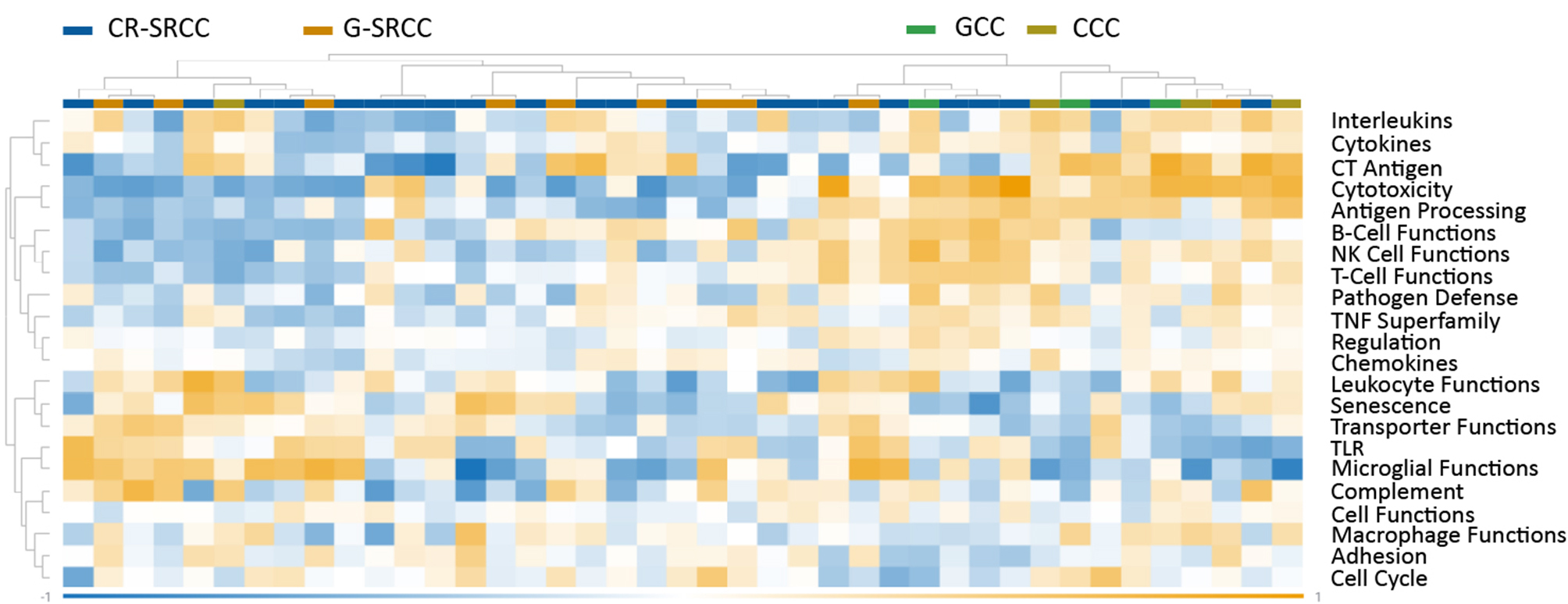

Supplement: Supplementary file 1 [file cells-15-00030-s001.zip › Supplemental Figure-S3_10022025 (1).jpg]

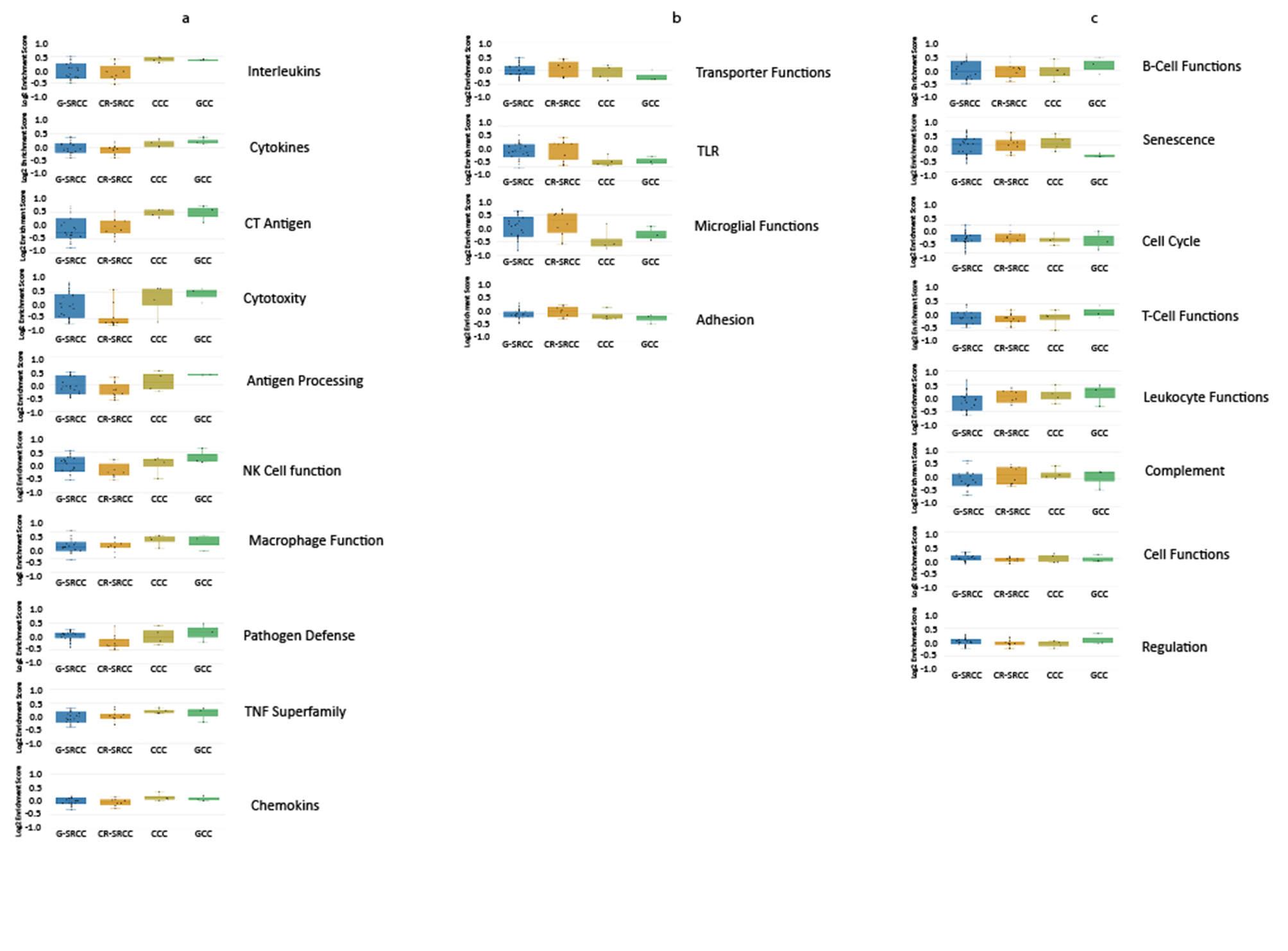

Supplement: Supplementary file 1 [file cells-15-00030-s001.zip › Supplemental Figure-S4_10022025 (1).jpg]
